# Relationship of neurocognitive ability, perspective taking, and psychoticism with hostile attribution bias in non-clinical participants: Theory of mind as a mediator

**DOI:** 10.3389/fpsyg.2022.863763

**Published:** 2022-08-31

**Authors:** Se Jun Koo, Ye Jin Kim, Eunchong Seo, Hye Yoon Park, Jee Eun Min, Minji Bang, Jin Young Park, Eun Lee, Suk Kyoon An

**Affiliations:** ^1^Section of Self, Affect and Neuroscience, Institute of Behavioral Science in Medicine, Yonsei University College of Medicine, Seoul, South Korea; ^2^Graduate Program in Cognitive Science, Yonsei University, Seoul, South Korea; ^3^Department of Psychiatry, Chonnam National University Medical School, Gwangju, South Korea; ^4^Yonsei Always Psychiatry Clinic, Seoul, South Korea; ^5^Department of Psychiatry, Yonsei University Wonju College of Medicine, Wonju Severance Christian Hospital, Wonju, South Korea; ^6^Department of Psychiatry, CHA Bundang Medical Center, CHA University, Seongnam, South Korea; ^7^Department of Psychiatry, Yonsei University College of Medicine, Yongin Severance Hospital, Yongin, South Korea; ^8^Department of Psychiatry, Yonsei University College of Medicine, Severance Hospital, Seoul, South Korea

**Keywords:** social cognition, attribution bias, psychoticism, perspective taking, theory of mind

## Abstract

**Objectives:**

Hostile attribution bias is reportedly common from non-clinical population to those with serious mental illness, such as schizophrenia, and is known to be closely related to theory of mind (ToM). This study aimed to investigate whether ToM skills mediate the relationship among neurocognitive ability, personality traits, and attribution bias.

**Methods:**

A total of 198 (101 females) non-clinical youths were recruited. To assess their neurocognitive ability and ToM skills, the participants were asked to complete Raven’s Standard Progressive Matrices (SPM) and the Korean version of the Reading the Mind in Eyes Test (K-RMET). To determine their personality traits, the Eysenck Personality Questionnaire (psychoticism) and interpersonal reactivity index (perspective taking) were used. To evaluate hostile attribution bias, the Ambiguous Intentions Hostility Questionnaire was administered. Path analysis and bias-corrected percentile bootstrap methods were used to estimate model fit and the parameters of the mediating effects.

**Results:**

Based on model comparison, the best model characterized (1) two direct pathways from psychoticism and the K-RMET to hostility attribution bias and (2) three indirect pathways, wherein SPM, perspective taking, and psychoticism influenced hostile attribution bias through K-RMET. The final model fit indices were good [*x*^2^/*df* = 1.126; comparative fit index = 0.996; root mean square error of approximation = 0.026; standard root mean square residual = 0.026 and Akaike information criterion = 28.251] and the K-RMET fully mediated the association between SPM, perspective taking, psychoticism, and hostile attribution bias.

**Conclusion:**

The main findings suggested that ToM skills, such as the RMET, play an important role in explaining the relationship among neurocognitive ability, personality traits, and hostile attribution bias. ToM skills and a remediation strategy may need to be developed to balance the enhanced hostility bias that underlies the paranoia.

## Introduction

When humans interact with others in social situations, it is important to have a clear understanding of the intentions of others. Social cognition is defined as the capacity to perceive and interpret social information on self and others. In social psychology, social cognition is the subject of research on specific phenomena related to social interaction such as stereotypes (Hamilton et al., 1994), Machiavellianism (Lyons et al., 2010), and altruism (Krebs and Van Hesteren, 1994), whereas in psychiatry, the concept of social cognition is applied to understand the characteristics of individuals experiencing mental disorders. Therefore, both psychiatrists and clinical psychologists have emphasized the importance of cognitive functions related to the processing of social information of individuals experiencing mental health difficulties (Mosiołek, 2014; Wyer and Srull, 2014). In schizophrenia research, social cognition is conceptualized in the following five domains (Green et al., 2008): Theory of mind (ToM), Social perception, Social knowledge, Attributional bias, and Emotional processing.

Attribution bias is defined as an individual’s interpretation of what caused a positive or negative outcome to occur. Among these, hostile attribution bias is referred to as the tendency to interpret the intentions of others as hostile despite the lack of the sufficient supporting information in certain situations (Milich and Dodge, 1984; Combs et al., 2007). Contrary to anger, which is known as an emotional response to aggressive behavior, hostile attribution biases are associated with cognitive schemas in which aggression is formed (Epps and Kendall, 1995; Helfritz-Sinville and Stanford, 2014), and if these biases are persistent and consolidated, they may develop into persecutory delusions (Kinderman and Bentall, 1996; McKay et al., 2005; An et al., 2010).

Theory of mind is defined as the ability to understand the states of mind, such as the intentions, beliefs, and desires of others (Premack and Woodruff, 1978). Around the age of 2 years, children begin to recognize some characteristics of their state of mind, and by the age of 4 or 5 years, most children can recognize that the mind is a representation (Wimmer and Perner, 1983; Baron-Cohen et al., 1985). To understand the mind state of another person, ToM requires the ability to interpret and reason about ambiguous situational and interpersonal details, such as the surrounding environment and facial emotions (Baron-Cohen et al., 2001). If ToM does not function properly, the possibility of external personalizing attribution rather than external situational attribution increases in an environment where the intentions of others are not clear (Taylor and Kinderman, 2002; Jeon et al., 2013). ToM deficit is also known to be closely related to mental disorders, such as autism and schizophrenia. In particular, in schizophrenia spectrum disorders, ToM deficit has been reported in not only full-blown psychosis but also prodromal phases, and it was reported to be a factor associated with an ultra-high risk for psychosis (Van Donkersgoed et al., 2015; S Vyas et al., 2017; van Neerven et al., 2021).

In addition to the aforementioned ToM deficits, there are other variables that can influence the relationship with hostile attribution bias. Neurocognition, which refers to the overall cognitive ability, including working memory, processing speed, and executive functions (Green et al., 2000), can be changed by an individual’s internal state. It is known that when attributions are made in a state of cognitive overload, external information requiring an understanding of the intentions, and behaviors of others can be ignored (Gilbert et al., 1988). Individuals may also find it easy to blame others rather than to generate alternative situational explanations that require cognitive effort (Langdon et al., 2006). As such, the level of neurocognition may influence attribution bias and ToM.

Personality traits can also influence the relationship between ToM and hostile attribution bias. “Psychoticism” is a term that was originally proposed by Eysenck to describe psychotic vulnerability (Eysenck and Eysenck, 1975). However, there have been studies that Eysenck’s “Psychoticism scale” should be distinguished from psychoticism, which is used as a term to describe mental vulnerability in a spectrum from non-clinical to positive symptoms of schizophrenia in psychopathology frameworks (Knežević et al., 2019). Recently, Eysenck’s psychoticism is supposed to be closely related to anger, vandalism, aggression and low agreeableness, which are key to the formation of hostility attribution (Wood and Newton, 2003; Carrasco et al., 2006). In addition, psychoticism is related to impolite, untrusting, and unfriendly behavior (Eysenck and Barrett, 2013); in particular, psychoticism is known to be strongly associated with low agreeableness (De Fruyt et al., 1993; Colledani et al., 2018). Considering that ToM also has accumulated empirical studies supporting the relevance of agreeableness (Nettle and Liddle, 2008; Allen et al., 2017), psychoticism may have a negative effect on ToM.

On the other hand, the term “empathy” refers to sharing the positive and negative emotional states of another person. Among them, “perspective taking domain of empathy” is considered a cognitive process of empathy, in that it is a process to consider the situation from the other person’s point of view (Hoffman, 1978; Davis, 1980). High levels of perspective-taking skills can reduce the likelihood of provocations from others, leading one to blame others and consequently inhibit the anger response when abundant information and resources are considered (Mohr et al., 2007). By looking at the relationship between ToM and perspective taking of empathy, ToM is distinguished from perspective taking of empathy in that it has multiple components including beliefs, intentions, desires, and emotions (Wellman and Liu, 2004), but both overlap in that they are based on understanding the perspectives of others (Shamay-Tsoory et al., 2010; Bora and Köse, 2016). Considering that previous studies have reported a strong relationship between ToM and perspective taking (Barnes-Holmes et al., 2004; Bensalah et al., 2016), if the perspective taking step is successful, an individual can accurately understand the other person’s state of mind, ultimately reducing the tendency to have a hostile attributing bias.

It is challenging to determine how to measure an individual’s ToM because the degree to which one understands others’ minds and intentions can vary according to context and circumstances. The reading the mind in eyes test (RMET) is one of the most widely used tests for measuring ToM. The test consists of a total of 36 photographs around the eyes, and participants are asked to choose words that best describe the intentions and emotions of the person in the photographs (Baron-Cohen et al., 2001). Since, the information provided to select the correct answer in the task is limited, the participants have to carefully examine each word and picture and distinguish nuances between the words. The RMET has been translated into various languages and is being used in the study of clinical populations with ToM deficit (Kettle et al., 2008; Bora et al., 2009; Peñuelas-Calvo et al., 2019). Recently, the Korean version of the RMET (K-RMET) was developed to overcome the limitation that the existing RMET may not reflect racial and cultural characteristics well, and in a study comparing the psychometric properties of the RMET and the K-RMET, acceptable reliability and validity were reported (Koo et al., 2021).

Given these findings, it can be inferred that ToM plays an important role in the relationship among variables that may influence hostile attribution bias, such as neurocognition and personality traits. However, there is little known about the mechanisms of this relationship and variables. This study aimed to investigate whether ToM skills mediate the relationship among neurocognitive ability, psychoticism, perspective taking, and hostile attribution bias using the K-RMET.

## Materials and methods

### Participants

A total of 198 healthy participants were recruited through online job advertisements. Using the mini international neuropsychiatric interview (MINI), nine psychiatrists and two clinical psychologists excluded participants with past or current psychiatric illnesses. In all, 14 participants were excluded because of incomplete data collection, leaving a final sample of 184 individuals. The final sample included 96 (51.9%) women and 88 (48.1%) men with a mean age of 23.02 years (SD = 2.63, range = 19–30). All research procedures were conducted in accordance with the tenets of Declaration of Helsinki and approved by the Institutional Review Board of the Severance Hospital (IRB No. 4-2014-0744). Informed consent was obtained from all the participants included in the study.

### Procedure

Each participant received a set of questionnaires, including the interpersonal reactivity index (IRI), the Korean version Eysenck Personality Questionnaire (K-EPQ), and the Korean version of the Ambiguous Intentions Hostility Questionnaire (K-AIHQ). Each participant performed the computerized standard progressive matrices (SPM) test and the K-RMET, which was examined by a psychologist (SJK and YJK) in a controlled laboratory. The tasks lasted for approximately 60–70 min.

### Measures

#### Ambiguous intentions hostility questionnaire

The Korean version of the K-AIHQ (Combs et al., 2007; Chang et al., 2009) comprises 15 vignettes of negative interpersonal situations, and the participants are asked to answer questions about how they would react in each situation. The questionnaire is divided into three situations (intentional situation, accidental situation, and ambiguous situation) with ambiguity of the intention. In the present study, five vignettes related to ambiguous situations were employed for analysis. In each vignette, the participants are asked to answer three types of questions related to attribution bias: hostility, blame, and aggression. For hostility and aggression, the participants’ answers to the open-ended question were evaluated by the rater on a Likert scale of 1–5 points (range from 10 to 50 points). To calculate inter-rater reliability, a subset of samples (*n* = 30) was randomly selected before determining the intraclass correlation coefficients [ICC] (3, 2); the ICCs of hostility (range from 0.915 to 0.965) and aggression (range from 0.878 to 0.927) indicated good to excellent correlation (Koo and Li, 2016). Blame bias was calculated as the average score of responses evaluated on a 5- or 6-point Likert scale (range from 15 to 80), which asks how much to blame and hold others accountable for the actions of others in each situation. The internal consistency (Cronbach’s alpha) was 0.88.

### Interpersonal reactivity index

The IRI (Davis, 1983; Kang et al., 2009) is a multidimensional scale that measures empathy tendencies and consists of four subscales of 28 items. The IRI originally comprised four dimensions: (1) perspective taking, (2) fantasy, (3) personal distress, and (4) empathic concern. In the present study, only the taking perspective subscale was used to focus on the cognitive empathy. Items were rated on a 5-point Likert scale, with 1 representing an inaccurate description of the participant and 5 representing a very accurate description (range from 5 to 35). The internal consistency (Cronbach’s alpha) was 0.64.

### Eysenck personality questionnaire

To evaluate the psychoticism personality trait, the psychoticism scale (P) of the K-EPQ (Eysenck and Lee, 1985; Lee, 1997) was used. A high score indicates coldness; lack of sympathy; and unfriendly, distrustful, odd, antisocial, and aggressive behavior. It consists of a total of 17 items, and participants were asked to respond to all items with “yes” (1 point) or “no” (0 points; range from 0 to 17). The internal consistency (Cronbach’s alpha) was 0.64.

### Standard progressive matrices

The SPM (Raven et al., 1990) is a non-verbal test was developed to measure neurocognitive reasoning by analogy. As a representative test to measure “reasoning and problem solving,” it has been used in various countries and cultures owing to its stable reliability and validity (Raven, 2000; Fett et al., 2011). The SPM comprises 60 non-colored diagrammatic puzzles, and the participants were asked to select the missing part of the presented matrix from among multiple options. The total score was calculated as the sum of the correct answers (range from 0 to 60).

### Reading the mind in the eyes

In the K-RMET (Koo et al., 2021), a photograph of a Korean person’s face was used based on the procedure in the original the RMET (Baron-Cohen et al., 2001). The same age (young, middle, elder), sex, and pupil orientation were applied as in the original version of the RMET, except for ethnicity. The test consists of 37 photographs of the eyes and their surrounding areas, including one practice question and the participant is asked to choose from among four options (one target, three foils) that would best describe what the person in the picture is thinking or feeling without a time limit. In the standardization study of the K-RMET and RMET, the K-RMET had acceptable reliability and validity. The total score was calculated as the sum of the correct answers (range from 0 to 36).

### Statistical analysis

Descriptive statistics and zero-order correlations among variables were calculated using the Statistical Package for the Social Sciences (SPSS), version 25 for Windows (IBM Corporation, Armonk, NY, United States). Path analysis was used to investigate potential pathways among neurocognition, psychoticism, ToM, and hostile attribution bias using AMOS 25 (IBM Corp., Armonk, NY, United States). All missing data were excluded, and the data were analyzed using maximum likelihood estimation. Model fit was measured based on conventional cut-offs [chi-square/degree of freedom ratio (*x*^2^/*df*) < 3], root mean square error of approximation (RMSEA) ≤0.08, standardized root mean squared residual (SRMR) ≤0.08, and comparative fit index (CFI) ≥0.9 (Hu and Bentler, 1999; Schermelleh-Engel et al., 2003; Schumacker and Lomax, 2016). The Akaike information criterion (AIC) was used for comparison between hypothetical models and model selection, and the model with the lowest AIC value was determined as the best fit model (Akaike, 1973; Burnham et al., 2011). Bias-corrected percentile bootstrapping with a 95% confidence interval (CI) was performed (*n* = 5,000) to estimate the parameters of mediating effects. All tests were two-tailed and conducted at 5% level of statistical significance.

## Results

### Preliminary analyses

Normality was tested for each variable before proceeding with further analysis. All variables were distributed within the criteria of skewness (∣skewness ∣ < 2) and kurtosis (∣kurtosis ∣ <7; Curran et al., 1996). Table 1 shows the means and standard deviations and Pearson’s correlations for all variables. Correlation analysis revealed that the total score of the K-RMET, which was used to measure the parameter of ToM, was significantly correlated with the scores of the SPM (*r* = 0.306, *p* < 0.001), psychoticism of K-EPQ (*r* = −0.250, *p* = 0.001), perspective taking of IRI (*r* = 0.236, *p* = 0.001), and AIHQ hostility ambiguous (*r* = −0.222, *p* = 0.002). Among the subscales of ambiguous situations in the AIHQ, the blame ambiguous and aggression ambiguous variables had no statistically significant correlation with most of the variables including K-RMET. For simplicity of the analysis, only the hostility ambiguous variable was selected as the criterion variable in further analysis.

**Table 1:** Descriptive statistics and correlation analysis of variables (*n* = 184).

	Mean	SD	1	2	3	4	5	6	7
1. SPM^a^	52.30	5.31	–						
2. Psychoticism	2.30	2.15	−0.146^*^	–					
3. Perspective taking	2.52	0.55	0.130	−0.314^***^	–				
4. K-RMET^b^	26.88	3.31	0.306^***^	−0.250^**^	0.236^**^	–			
5. AIHQ^c^ blame ambiguous	2.41	0.67	−0.119	0.143	−0.233^**^	−0.094	–		
6. AIHQ hostility ambiguous	1.68	0.60	−0.173^*^	0.199^**^	−0.130	−0.222^**^	0.604^***^	–	
7. AIHQ aggression ambiguous	1.51	0.32	−0.122	−0.026	−0.131	0.024	0.177^*^	0.068	–

a Standard Progressive Matrices,

b Korean version of the RMET,

c Korean version of the Ambiguous Intentions Hostility Questionnaire.

* *p* < 0.05;

** *p* < 0.01;

*** *p* < 0.001.

### Path analysis

Prior to main study, to confirm the direction of the mediating effect, the pathway model using psychoticism and perspective taking as a mediator, respectively, and the model using ToM as a mediator were compared. When the path analysis was conducted, the three models had equal model fit indices (Supplementary Table 1), but the path between the mediator and hostile attribution bias was not statistically significant in other models except ToM-mediated model (Supplementary Figure 1).

**Figure 1:**
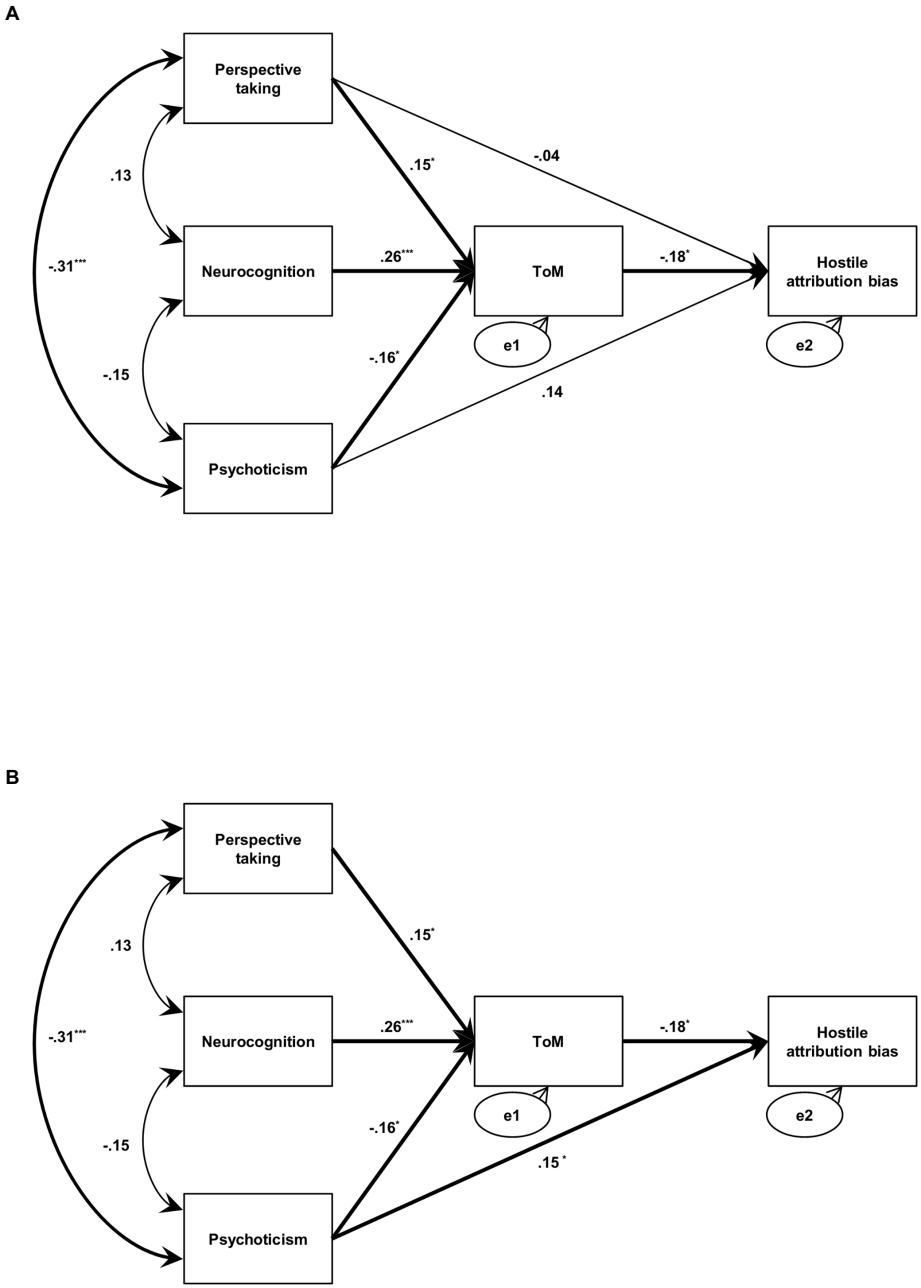
Basic model showing ToM as a mediator of the relationship. **(A)** Modified model. **(B)** ^*^p < 0.05; ^**^p < 0.01; ^***^p < 0.001. Single-headed arrows indicate standardized regression weights; double-headed arrows indicate correlations. ToM, Theory of Mind.

The results of the path analysis performed for the two models are presented in Figure 1. The first model was named “basic model,” wherein ToM mediated all exogenous variables plus psychoticism and perspective taking variables with a direct path toward hostile attribution bias (Figure 1A). Since, the correlation between exogenous variables was significant in Pearson’s correlation, the correlation pathways of all exogenous variables were also added to the path model. In this model, all model fit indices were at an adequate to good level (*x*^2^/*df* = 1.913; CFI = 0.985; RMSEA = 0.071; SRMR = 0.024 and AIC =29.913). While all other pathways were significant, the direct pathways from psychoticism to hostile attribution bias (ß = 0.141, *p* = 0.064) and from perspective taking to hostile attribution bias (ß = −0.044, *p* = 0.561) were not significant.

The “modified model” excluded the direct path from perspective taking to hostile attribution bias in the basic model (Figure 1B). In this model, all model fit indices were at an adequate level (*x*^2^/*df* = 1.126; CFI = 0.996; RMSEA = 0.026; SRMR = 0.026 and AIC = 28.251), and all direct paths including psychoticism to hostile attribution bias (ß = 0.154, *p* = 0.037), neurocognition to ToM (ß = 0.262, *p* < 0.001), psychoticism to ToM (ß = −0.164, *p* = 0.022), perspective taking to ToM (ß = 0.150, *p* = 0.036), and ToM to hostile attribution bias (ß = −0.184, *p* = 0.012) were also significant. As shown in Table 2, the modified model was also at a good level in all model fit indices, and the modified model in AIC, which considered both model fit and simplicity, was better than the basic model. Therefore, the modified model was selected as the final model.

**Table 2:** Model comparison.

	*x* ^2^	*df*	*p*	*x*^2^/*df*	CFI^a^	RMSEA^b^	SRMR^c^	AIC^d^
Basic model	1.913	1	0.167	1.913	0.985	0.071	0.024	29.913
Modified model	2.251	2	0.324	1.126	0.996	0.026	0.026	28.251

a Comparative fit index.

b Root mean square error of approximation.

c Standardized root mean squared residual.

d Akaike information criterion.

### Mediation analysis

Based on the aforementioned final model, mediation effects were calculated using direct and indirect effects based on a bootstrap procedure (*n* = 5,000) and bias-corrected bootstrap and are presented in Table 3. As a result of the bias-corrected percentile bootstrap, all indirect effects, including hostile attribution bias from psychoticism [ß = 0.030, CI (0.001, −0.097), *p* = 0.032], hostile attribution bias from neurocognition [ß = −0.048, CI (−0.139, −0.004), *p* = 0.023], and hostile attribution bias from perspective taking [ß = −0.028, CI (−0.083, −0.001), *p* = 0.035], were statistically significant. The direct effects from psychoticism to ToM [ß = −0.164, CI (−0.311, −0.017), *p* = 0.028], from neurocognition to ToM [ß = 0.262, CI (0.080, 0.437), *p* = 0.006], from perspective taking to ToM [ß = 0.150, CI (0.020, 0.291), *p* = 0.025] and from ToM to hostile attribution bias [ß = −0.184, CI (−0.354, −0.009), *p* = 0.037] were also significant, whereas the direct path from psychoticism to hostile attribution bias was not significant [ß = 0.154, CI (−0.033, 0.363), *p* = 0.105].

**Table 3:** Direct and indirect effect of variables (bootstrap = 5,000).

	Psychoticism				Perspective taking				SPM^a^				ToM^b^			
	Direct effect		Indirect effect		Direct effect		Indirect effect		Direct effect		Indirect effect		Direct effect		Indirect effect	
	Estimate^c^	*p*	Estimate	*p*	Estimate	*p*	Estimate	*p*	Estimate	*p*	Estimate	*p*	Estimate	*p*	Estimate	*p*
ToM	−0.164	0.028	–	–	0.150	0.025	–	–	0.262	0.006	–	–	–	–	–	–
Hostility ambiguous	0.154	0.105	0.030	0.032	–	–	−0.028	0.035	–	–	−0.048	0.023	−0.184	0.037	–	–

a Standard progressive matrices,

b Theory of mind,

c Standardized regression weights.

## Discussion

To our best knowledge, this is the first study to evaluate the role of ToM as a mediator among neurocognition, psychoticism, perspective taking, and hostile attribution bias in the non-clinical population. As expected, ToM showed a significant mediating effect in the final model derived from this study. More specifically, the final model had better AIC than the basic model as well as the competitive model in which other variables used in the study, such as psychoticism and perspective taking, were employed as mediators, respectively, and was good in all model fit indices. As shown in the final model (Figure 1B), all standardized regression coefficients for each path were also significant. Interestingly, in the bias-correction bootstrapping performed to analyze the direct and indirect effects, the direct effect of psychoticism was not significant, while all the indirect pathways toward hostile attribution bias were significant. These results suggested that ToM fully mediated the relationship between hostile attribution bias and other exogenous variables.

Neurocognition, as indexed by SPM, showed a positive relationship with ToM, and this result is consistent with a recent study that reported the relationship between cognitive ability and the RMET (Baker et al., 2014; Peñuelas-Calvo et al., 2019). On the other hand, the results of previous studies on the relationship between neurocognition and hostile attribution bias are mixed, wherein some studies showed that individuals with high neurocognitive abilities made few hostile attribution biases (Choe et al., 2013; Lee et al., 2019), whereas other studies have reported that there was no significant correlation between neurocognitive ability and hostile attribution bias (Jeon et al., 2013; Sorge et al., 2015). Since, previous studies investigated only the direct relationship between the two variables described above, we built an extended model by adding ToM as a mediator. In this study model, neurocognition was found to predict hostile attribution bias through a ToM-mediated pathway. The K-RMET employed to measure ToM in this study required considerable cognitive effort to choose the correct answer because limited information related to the face (around the eyes) was presented (Baron-Cohen et al., 2001). Therefore, in a situation where the cognitive state is affected by the external environment, the mechanism for clearly understanding the internal state of the other person is hampered, and in the end, it may not be possible to accurately attribute the intention of the other person.

It is known that personality traits are closely related to an individual’s attribution style (Furnham, 2009; O’Donnell et al., 2013). In this study, among personality traits, psychoticism and perspective taking were employed to achieve an integrated understanding of various variables affecting hostile attribution bias. In our results, psychoticism and perspective taking showed a negative correlation, and they played opposite roles in the relationship between ToM and hostile attribution bias within the study model. Psychoticism and hostile attribution bias share characteristics in that they are temperament and cognitive errors, respectively, that “increase the likelihood of aggression,” and a significant relationship has been reported in previous studies as well (da Costa et al., 2018). In the presence of psychoticism, including hostile, untrusting, and rude behavior and lack of human feelings, the desire or need to understand the other person’s feelings and intentions is very low (Mobini et al., 2006), and even in ambiguous situations, attribution bias can easily occur. Conversely, individuals with high perspective-taking skills of empathy can infer the thoughts or beliefs of another agent (Healey and Grossman, 2018) and are likely to understand the other’s mental state more accurately than those without such skills (Barnes-Holmes et al., 2004); hence, the formation of a hostile attribution bias can be prevented. Our findings serve as evidence supporting that psychoticism and perspective taking may act as risk factors and protective factors, respectively, against illogical and persistent hostile attribution bias, which may develop into persecutory delusions.

In addition to main findings related to path analysis, this study provides two important pieces of information related to measurement tools. First, in some studies on the psychometric properties of AIHQ, there was a problem with its reliability because the hostility index was evaluated by the raters (Buck et al., 2017). However, in our data, ICC indicated good to excellent results (range from 0.915 to.965), and the hostility index was significantly associated with other variables used in the present study. Since, the hostility index is a starting point and a core cognitive aspect of the “hostile” attribution bias, if the ICC can be improved through sufficient training between raters, it can be a reliable and valid tool for measuring attribution bias. Second, the K-RMET employed to measure ToM in this study significantly mediated the related variables. This suggests that the RMET is a reliable test tool to measure ToM. In particular, when ToM needs to be measured through a small number of tests under limited time and conditions, the RMET (including the K-RMET), which is known to reflect the cognitive and affective characteristics of ToM, may be one of the best options.

The present study had certain limitations. First, since participants in early adulthood or late adolescence were recruited in the present study, the application of these findings to all age groups is limited. Second, only SPM was used for neurocognitive measurements. In future research, a measurement tool that covers various areas of neurocognition should be used to provide a more comprehensive view of the results of this study. Third, there are studies that the RMET is suitable to measure socio-perceptual ToM (Nettle and Liddle, 2008; Mitchell and Phillips, 2015), and since ToM itself is a multiple domain, if follow-up studies are conducted by constructing the same model with neurocognition, psychoticism, perspective taking, and hostile attribution bias with other tests such as The Awareness of Social Inferences Test (McDonald et al., 2003) and Hinting Task (Corcoran et al., 1995) developed to measure ToM instead of the RMET, it could be helpful to understand the relationship between related variables. Last, since this study used cross-sectional data, there are limitations in drawing firm conclusions about the causal relationship among related variables. A longitudinal study is needed to exclude various confounding factors.

In summary, our data showed that neurocognitive ability, psychoticism and perspective taking affected that ToM, which was associated with hostile attribution bias through direct and indirect paths. In ToM, one of the main domains of social cognition, plays an important mediating role in the relationship among attribution bias, another domain of social cognition, and other related variables, it is possible that the relationship among domains of social cognition may be parallel and influence each other at the same time. In future studies, it will be worthwhile to explore whether the relationship between the aforementioned attribution styles and ToM and other relevant variables might also be observed in clinical populations such as those with schizophrenia and autism spectrum disorders who persistently present ToM deficits.

## Data availability statement

The raw data supporting the conclusions of this article will be made available by the authors, without undue reservation.

## Ethics statement

The studies involving human participants were reviewed and approved by Institutional Review Board of the Severance Hospital. The patients/participants provided their written informed consent to participate in this study.

## Author contributions

SA: conceptualization. YK, SK, JM, ES, and HP: data curation. SK and YK: formal analysis. SA, YK, SK, JM, ES, and HP: investigation and writing—review & editing. SK and MB: methodology. SA and EL: project administration and resources. SK: software and writing—original draft. SA: supervision. MB, ES, HP, and JP: validation. All authors contributed to the article and approved the submitted version.

## Funding

This work was supported by the Basic Science Research Program through the National Research Foundation of Korea (NRF) funded by the Ministry of Science, ICT & Future Planning, Republic of Korea (grant number: 2017R1A2B3008214 and 2022R1A2B5B03002611).

## Conflict of interest

The authors declare that the research was conducted in the absence of any commercial or financial relationships that could be construed as a potential conflict of interest.

## Publisher’s note

All claims expressed in this article are solely those of the authors and do not necessarily represent those of their affiliated organizations, or those of the publisher, the editors and the reviewers. Any product that may be evaluated in this article, or claim that may be made by its manufacturer, is not guaranteed or endorsed by the publisher.
