# Supplementary material for: Relationship of neurocognitive ability, perspective taking, and psychoticism with hostile attribution bias in non-clinical participants: Theory of mind as a mediator
Source: Front Psychol. 2022 Aug 31;13:863763. doi: 10.3389/fpsyg.2022.863763 (PMC9471867; doi:10.3389/fpsyg.2022.863763)
Supplement: Supplementary file 1 [file Data_Sheet_1.PDF]

Supplementary Material

1 **Supplementary Table 1.** Competitive model comparison

	x^2	*df*	*p*	x^2/df	CFI[a]	RMSEA[b]	SRMR[c]	AIC[d]
Perspective taking mediated	1.913	1	.167	1.913	.985	.071	.024	29.913
Psychoticism mediated	1.913	1	.167	1.913	.985	.071	.024	29.913
ToM[e] mediated	1.913	1	.167	1.913	.985	.071	.024	29.913

[a]Comparative Fit Index, [b]Root Mean Square Error of Approximation, [c]Standardized Root Mean Squared Residual, [d]Akaike Information Criterion, [e]Theory of Mind.

2 Supplementary Figure Legends

Supplementary Figure 1. Perspective taking mediated model (A), Psychoticism mediated model (B), ToM mediated model (C).
$^{*}p < 0.05$; $^{**}p < 0.01$; $^{***}p < 0.001$.
Note. Single-headed arrows indicate standardized regression weights; double-headed arrows indicate correlations. ToM = Theory of Mind.
