# Supplementary figures and images for: Relationship of neurocognitive ability, perspective taking, and psychoticism with hostile attribution bias in non-clinical participants: Theory of mind as a mediator
Source: Front Psychol. 2022 Aug 31;13:863763. doi: 10.3389/fpsyg.2022.863763 (PMC9471867; doi:10.3389/fpsyg.2022.863763)

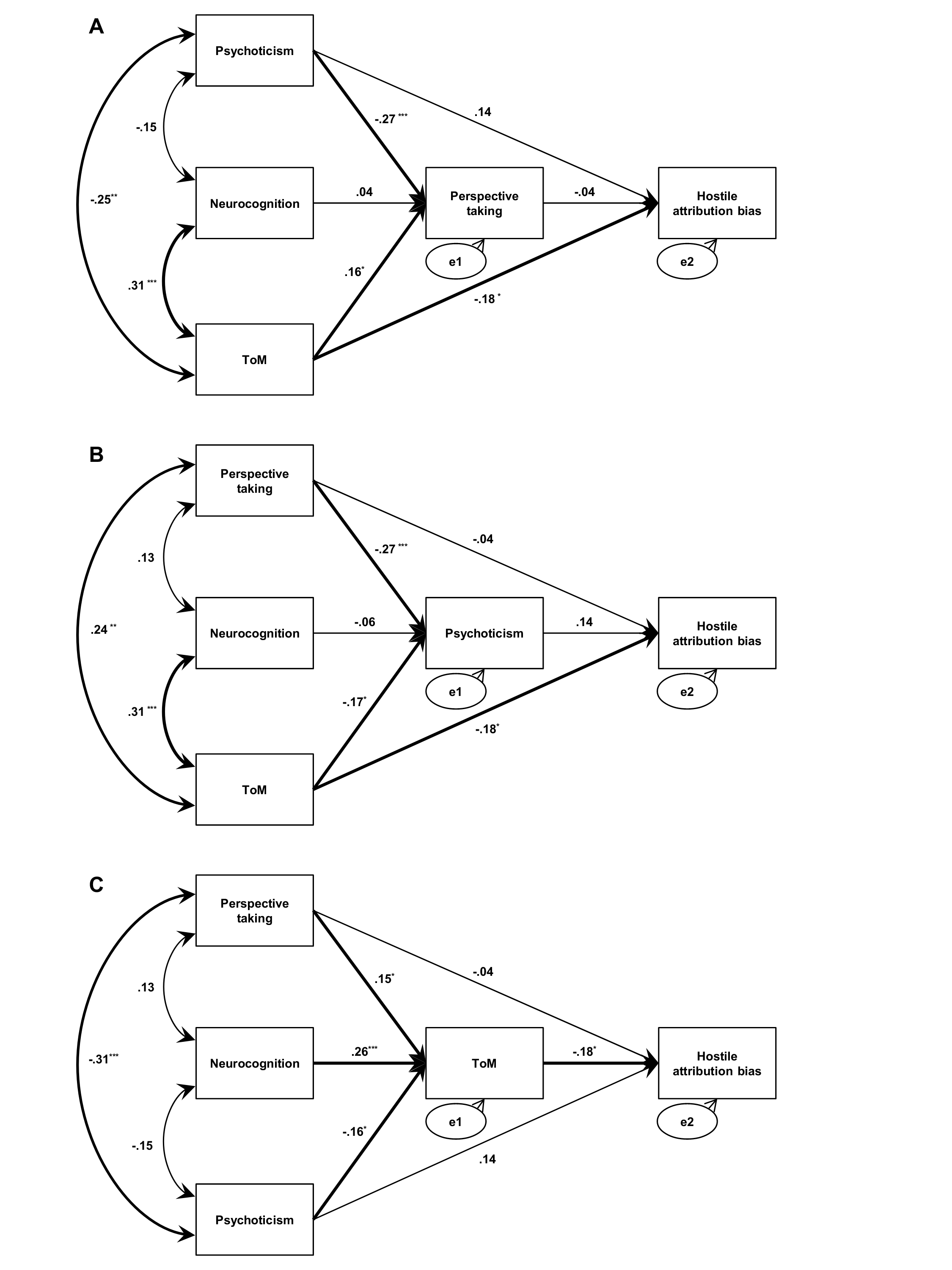

Supplement: Supplementary file 2 [file Image_1.TIFF]
